# Expansion of *Anopheles maculipennis* s.s. (Diptera: Culicidae) to northeastern Europe and northwestern Asia: Causes and Consequences

**DOI:** 10.1186/1756-3305-7-389

**Published:** 2014-08-22

**Authors:** Yuri M Novikov, Oleg V Vaulin

**Affiliations:** Department of Cytology and Genetics of Tomsk State University, Tomsk, Russia; Institute of Cytology and Genetics SB RAS, Novosibirsk, Russia

**Keywords:** Global warming, Changes in distribution areas, Cryptic species, *Anopheles maculipennis*, *Anopheles messeae* s.l, *Anopheles beklemishevi*, Cytogenetics, ITS2 PCR-RFLP, Ecological niche

## Abstract

**Background:**

The burden of malaria infection in the modern world remains significant. Specific changes in the relative proportions of malaria vector mosquitoes, Maculipennis Complex species, in the south of Western Siberia over the past 25 years of the 20^th^ century have attracted wide attention as an indicator of their dynamic geographical distribution. In Eurasia, studies of fluctuations in the borders of areas occupied by sibling species of this complex, as well as their relative proportions in the areas where they are sympatric are epidemiologically important.

**Methods:**

Species identity and chromosomal polymorphisms within each population were defined by cytogenetic analysis of polytene chromosomes of third- and fourth-instar larvae and adult females of *Anopheles* mosquitoes collected over the period from 1973 to 2012. A total of 37 *Anopheles* samples (3,757 specimens) from the Ukraine, European Russia and the Urals were studied. To identify *An. messeae* s.l. cryptic species A and B, polymerase chain reaction and restriction fragment length polymorphisms of the second internal transcribed spacer rRNA genes sequences (ITS2 PCR-RFLP) were used.

**Results:**

*An. maculipennis* s.s*.* is expanding to the northeast at a speed of approximately 30 km per year. In 2008 or 2009, this species appeared in the Southern Urals. The emergence of *An. maculipennis* in this region was accompanied by a decrease in the proportions of *An. messeae* A and *An. beklemishevi* and by an increase in the proportion of *An. messeae* B within *An. messeae* s.l. It is highly likely that the southwestern border of *An. beklemishevi* distribution area could shift in the same direction as expanding area of *An. maculipennis*.

**Conclusions:**

The geographical distribution of the Palaearctic mosquito species of the Maculipennis Complex is undergoing a gradual shift. Changes detected in the species distribution can be considered as a component of the biocenotic process triggered by global warming. Both the warming itself and consequent expansion of *An. maculipennis* s.s. to the northeast, followed by changes in the species composition of *Anopheles* as well as their relative proportions and fluctuations in the species areas, exacerbate the epidemiology of malaria infection in Eurasia.

## Background

Malaria remains a serious health issue in the contemporary world, being one of the major causes of death from infectious diseases worldwide. Global epidemiological situation in relation to malaria spread remains complicated [1, 2]. The complexity and danger of the situation are aggravated by progressive global warming [3]. Manson [4] pointed out the direct role of temperature on the spread of malaria infection. Its indirect role is also significant, as temperature affects the duration of life cycle both of disease agents and vectors [5, 6]. Despite being closely related, species of the *Anopheles* family differ in their physiology and ecology, including temperature sensitivity. Thus, changes in regional temperatures can influence the geographical distribution of *Anopheles* species as well as their interactions in the regions where the species are sympatric. Due to variable abilities of different species for transmission of malaria [7–9], global warming can result in profound changes in the risk of malaria infection in many regions of the world. Understandably, with a rise of annual average temperature the duration of warm time of the year will increase, making it possible for southern, i.e. most effective, *Plasmodium* transmitters [7] to invade the north of Eurasia. At the time when global warming had been admitted to be a serious issue, the borders of areas occupied by sibling species within *Anopheles maculipennis* Mg. taxon [7, 8, 10–12] were hardly defined [13–16]. In light of the above facts, refinement of these borders and analysis of their dynamics is becoming increasingly important. Climate projections can be used to predict dynamics of the borders [17, 18]; in a number of studies both actual changes and likely future changes of the borders were reported [8, 19–22]. Diverse responses to global warming are observed in different groups of species [23–25]. We believe that the present study, which was aimed to address the dynamics of the northeastern border of *Anopheles maculipennis* s.s. distribution area, will provide important insights into the role of global climate change in geographical distribution of the Palaearctic mosquito species of the Maculipennis Complex and its disease-relevant consequences.

Lack of consensus in views on the structure of *An. messeae* taxon presents a major obstacle in understanding of speciation-related events within Palaearctic Maculipennis Complex. Based on non-random combinations of chromosome inversions in natural populations as well as assortative mating and different ecological features, as early as in 1984 it was suggested that *An. messeae* includes two cryptic species. These were provisionally named *An. messeae* A and *An. messeae* B [26]. The existence of these two species was confirmed by taxonoprint DNA analysis [27]. Some researchers [22, 28] still regard *An. messeae* as a single polytypic, polymorphic species. Another research group isolated a new species, *An. daciae,* from *An. messeae* [29]. Based on the results of molecular-genetic research, *An. daciae* appeared to be identical to *An. messeae* A [30]. For this reason, in the present study the latter *An. messeae* cryptic species will be referred to as *An. messeae* A. *An. messeae* s.l. will therefore refer to both cryptic species A and B.

## Methods

Third and fourth instar *Anopheles* larvae and adult females were used. Locations, time of collection and relevant numerical data are given in Table 1. Larvae were obtained from still or partially flow-through fresh water reservoirs, as well as a brackish water lake (Muldakkul, located 15 km to the west of Magnitogorsk, Ozernoe, Republic Bashkortostan). Imagoes were collected in cattle barns. Larvae were fixed in Clark’s solution (100% ethanol and glacial acetic acid 3:1) or 96% ethanol. Squash preparations of salivary glands of larvae and Malpighian tubules of adult females were prepared as described elsewhere [31, 32]. Species identification and analysis of chromosomal inversions in *An. messeae* karyotypes were performed using polytene chromosomes maps described in [14, 33]. Determination of species composition in the populations was reported in part previously [34]. The inversion variants of polytene chromosomes were traditionally marked by indicating the polymorphic chromosome element of the polytene complex (1L, 2R) and its variants [33]. Variants 1L/1L, 1L/1L1, 1L1/1L1 of the left arm of chromosome 1 (sex, or X-chromosome) in females are homozygous standard, heterozygous and homozygous inverted sequences, respectively. Variants 1L/¬ and 1L1/¬ in males are hemizygous standard and inverted sequences, respectively. The 2R element in both sexes can be present in the following combinations: 2R/2R, 2R/2R1 and 2R1/2R1 – homozygous standard, heterozygous and homozygous inverted sequence, respectively. *An. messeae* species A and B have almost completely overlapping inversion polymorphisms and can be identified by inversions on sex chromosomes; they also differ quantitatively, by the frequencies of autosomal inversions [26, 27]. If a specimen possesses a combination of 1L1/1L1, 1L/1L1 or 1L1/¬ with the standard 2R version in its karyotype, it can be ascertained, with a probability close to 1, that this specimen belongs to *An. messeae* A. The variants 1L/1L1, 1L1/1L1 and 1L1/¬ almost never occur in the karyotype of *An. messeae* B. 2R/2R1 heterozygotes and 2R1/2R1 homozygotes almost always belong to *An. messeae* B [26, 27]. The results of cytogenetic analysis of *An. messeae* s.l. populations are summarized in Table 2. The frequencies of homozygotes 1L1/1L1 and heterozygotes 1L/1L1 (calculated in subpopulations of females and shown together), and homozygous 2R/2R allow us to determine the approximate proportions of *An. messeae* A in the populations studied. While the frequencies of homozygotes 2R1/2R1 and heterozygotes 2R/2R1 (so as for 2R/2R calculated in whole populations and shown together) allow us to estimate the proportions of *An. messeae* B. Thus, analysis of polytene chromosomes allows species identification only for those specimens which possess marker combinations of chromosome variants in their karyotype. *An. messeae* species A and B can be accurately identified by the taxonoprint analysis [27] or by patterns of PCR product digestion of the ITS2 by the *Bst*F5I restriction enzyme [30, 35]. The identities of 102 *An. messeae* s.l. specimens collected in Miass in 2012 were established by ITS2 PCR-RFLP.

**Table 1:** **Species composition of** ***Anopheles*** **samples**

Collection localities (coordinates)	Collection date	Number of specimens/***An.***species		
		***maculipennis***	***messeae***s.l.	***beklemishevi***
1 **Rybinsk** (58°03′N; 38°50´E)*	25.06.87	0	96	0
2 **Yaroslavl** (57°37′N; 39°51′E)*	15.07.87	0	88	0
3 **Rostov −**1 (57°11′N; 38°24′E)	21.07.87	0	165	0
**Rostov −**1*	24.07.87	3	140	0
**Rostov −**2*	20.08.87	5	118	0
4 **Solnechnogorsk** (56°11′N; 36°59′E)*	02.06.79	44	77	1
**Solnechnogorsk**	30.08.79	45	56	0
**Solnechnogorsk**	09.06.81	16	57	0
**Solnechnogorsk**	09.07.81	9	87	0
**Solnechnogorsk**	30.08.81	14	14	0
5 **Suzdal** (56°26′N; 40°26′E)*	02.06.79	6	28	2
6 **Protvino**, 54°52′N; 37°13′E	10.06.81	60	9	0
Protvino*	08.07.81	52	24	0
Protvino*	28.08.81	58	79	0
7 **Murom** (55°34′N; 42°02′E)*	28.08.86	81	5	0
8 **Verbovskii** (55°31′N; 41°59′E)*	27.08.86	10	190	2
9 **Yoshkar-Ola** (56°38′N; 47°53′E)*	02.09.86	2	178	0
10 **Zelenodolsk** (55°51′N; 48°31′E)*	07.08.73	0	118	1
Zelenodolsk*	04.06.79	2	15	0
11 **Samara** (53°11′N; 50°07′E)*	10.09.86	23	108	0
12 **Yablonovsky** (44°55′N;38°56′E)	27.08.81	0	78	0
13 **Khadyzhensk** (44°26′N; 39°22′E) LD	26.08.81	59	36	0
**Khadyzhensk** HD	26.08.81	86	26	0
14 **Kharkov** (50°00′N; 36°13′E)	22.08.98	93	18	0
15 **Kundravy** (54°50′N; 60°13′E)	16.09.81	0	100	0
16 **Miass**-1 (55°01′N; 60°06′E)	10.08.99	0	124	0
**Miass**-2 (55°00′N; 60°04′E)	12.08.99	0	126	0
**Miass**-1	25.08.00	0	157	0
**Miass**-1	26.08.10	1	137	0
**Miass**-1	16.08.11	2	28	0
**Miass**-3 (55°02′N; 60°06′E)	17.08.11	4	76	0
**Miass**-1	14.08.12	5	139	0
**Miass**-3	16.08.12	0	47	0
**Miass**-4 (54°58′N; 60°05′E)	20.08.12	0	101	0
17 **Ozernoe** (53°27′N; 58°47′E)	15.08.12	2	6	0
18 **Koltashi** (57°24′N; 60°52′E)	05.07.12	0	112	3
**Koltashi**	11.07.12	0	91	12

Note: * – published previously [33]; LD and HD – low and high larval density, respectively (a three-fold difference).

**Table 2:** **Dynamics of chromosome variants in** ***An. messeae*** **s.l., depending on the presence and relative abundance of** ***An. maculipennis***

Collection localities and dates	N	***An. maculipennis***frequency (%)	Frequency of chromosome variants in ***An. messeae***s.l. (%)		
			1L/1L1, 1L1/1L1	2R/2R	2R/2R1, 2R1/2R1
**Rybinsk** (25.06.87)	96 lv	0	86.5 ± 4.7	63.5 ± 4.9	36.5 ± 4.9
**Yaroslavl** (15.07.87)	88 lv	0	56.2 ± 7.2	60.2 ± 5.2	39.8 ± 5.2
**Rostov-1** (21.07.87)	165 lv	0	73.0 ± 4.7	61.2 ± 3.8	38.8 ± 3.8
**Rostov-1** (24.07.87)	143 lv	2.1 ± 1.2	85.9 ± 3.8	77.9 ± 3.5	22.1 ± 3.5
**Rostov-2** (20.08.87)	123 lv	4.1 ± 1.9	54.8 ± 5.8	62.7 ± 4.5	37.3 ± 4.5
**Solnechnogorsk** (02.06.79)	122 f	36.1 ± 4.3	20.8 ± 4.6	35.1 ± 5.4	64.9 ± 5.4
**Solnechnogorsk** (09.06.81)	73 f	21.9 ± 4.8	44.8 ± 6.6	55.3 ± 6.6	44.7 ± 6.6
**Solnechnogorsk** (09.07.81)	96 f	9.4 ± 3.0	42.5 ± 5.3	51.7 ± 5.4	48.3 ± 5.4
**Solnechnogorsk** (30.08.81)	28 f	50.0 ± 9.4	14.3 ± 9.4	35.7 ± 12.8	64.3 ± 12.8
**Suzdal** (02.06.79)	36 f	16.7 ± 6.2	17.8 ± 7.2	3.6 ± 3.5	96.4 ± 3.5
**Protvino** (28.08.81)	137 fd	42.3 ± 4.2	17.8 ± 4.3	67.1 ± 5.3	32.9 ± 5.3
**Verbovskii** (27.08.86)	202 lv	5.0 ± 1.5	7.7 ± 2.6	51.6 ± 3.6	48.4 ± 3.6
**Yoshkar-Ola** (02.09.86)	180 lv	1.1 ± 0.8	2.4 ± 1.4	34.1 ± 3.6	65.9 ± 3.6
**Zelenodolsk** (07.08.73)	119 lv	0	1.0 ± 1.1	43.2 ± 4.6	56.8 ± 4.6
**Samara** (10.09.86)	131 lv	17.6 ± 3.3	9.4 ± 4.0	96.3 ± 1.8	3.7 ± 1.8
**Yablonovsky** (27.08.81)	78 lv	0	70.0 ± 7.2	100	0
**Khadyzhensk** (26.08.81), LD	95 lv	62.1 ± 5.0	71.4 ± 9.9	100	0
**Khadyzhensk** (26.08.81), HD	112 lv	76.8 ± 4.0	57.1 ± 13.2	100	0
**Kundravy** (16.09.81)	100 fd	0	76.0 ± 4.3	95.0 ± 2.2	5.0 ± 2.2
**Miass-1** (10.08.99)	124 lv	0	56.3 ± 6.2	87.9 ± 2.9	12.1 ± 2.9
**Miass-2** (12.08.99)	126 lv	0	58.1 ± 6.3	90.5 ± 2.6	9.5 ± 2.6
**Miass-1** (25.08.00)	157 lv	0	61.8 ± 4.8	95.5 ± 1.7	4.5 ± 1.7
**Miass-1** (26.08.10)	138 lv	0.7 ± 0.7	44.9 ± 5.6	89.8 ± 2.6	10.2 ± 2.6
**Miass-1** (16.08.11)	30 lv	6.7 ± 4.6	40.0 ± 12.6	85.7 ± 6.6	14.3 ± 6.6
**Miass-3** (17.08.11)	80 lv	5.0 ± 2.4	19.1 ± 6.1	81.6 ± 4.4	18.4 ± 4.4
**Miass-1** (14.08.12)	144 lv	3.5 ± 1.5	34.2 ± 5.3	79.9 ± 3.4	20.1 ± 3.4
**Miass-3** (16.08.12)	47 lv	0	33.3 ± 8.6	91.5 ± 4.1	8.5 ± 4.1
**Miass-4** (20.08.12)	101 lv	0	32.1 ± 6.4	79.2 ± 4.0	20.8 ± 4.0
**Koltashi** (05.07.12)	115 lv	0	42.1 ± 6.5	62.5 ± 4.6	37.5 ± 4.6
**Koltashi** (11.07.12)	103 lv	0	40.5 ± 6.3	51.6 ± 5.2	40.4 ± 5.2

Note: lv – larvae, f – breeding females, fd – females in diapause; N – sample size; standard deviation is given as a measure of statistical error.

## Results and discussion

Analysis of species composition from different regions and years (Table 1) revealed the following:

1. The proportion of *An. maculipennis* in samples collected at approximately the same time increased from the north and east to the south and west, and ranged from 0% in the Middle Urals to 80% in the North Caucasus and the Ukraine.
2. *An. beklemishevi* was identified in the samples collected mostly in the northern and eastern regions, and its peak frequency was detected at the extreme northeastern location (Koltashi, 80 km north of Yekaterinburg, the Rezh river backwater).
3. The proportions of sibling species may differ in samples collected simultaneously in closely situated locations, yet from different biotopes (*An. maculipennis* and *An. messeae* in Yablonovsky and Khadyzhensk, Murom and Verbovsky); they also can fluctuate significantly during the same breeding season and even over several days within the same location and biotope (Solnechnogorsk, Protvino, Rostov, Koltashi).
4. In eastern regions (Zelenodolsk, Miass), *An. maculipennis* was absent in the earlier samples, but present in samples collected several years later.
5. *An. maculipennis*, *An. messeae* s.l. and *An. beklemishevi* may reside in the same biotope, as may *An. maculipennis* and *An. messeae* s.l. or *An. messeae* s.l. and *An. beklemishevi*; however, *An. maculipennis* and *An. beklemishevi* do not occur together in the absence of *An. messeae* s.l.

Elements of these observations are in agreement and show ecological diversification of the species at different stages of individual development. In particular, combinations of co-inhabiting species indicate that the ecological niche of *An. messeae* s.l. is the widest among species studied. Borders of areas occupied by *An. maculipennis* and *An. beklemishevi* most likely reflect their responses to abiotic factors, which are commonly changing geographically. Furthermore, competition between the latter two species during larval development is higher compared to competition of each of them with *An. messeae* s.l.

Analysis of mosquito larvae collected in different parts of the Pshish river backwaters, spaced by 5–6 m and characterized by variable population density (Table 2, Hadyzhensk), shows a prevalence of *An. maculipennis* in both zones, with a significantly higher proportion in the area with a higher population density (p < 0.05). This could be interpreted as a result of species-specificity in egg-laying sites of *An. maculipennis* that can give rise to microlocalities with higher density of this particular species. Another plausible scenario is active larval migration in accordance with ecological preferences. In either case, the specificity of distribution is due to the behavioral characteristics. The key factor here is how behavioral reactions of each species reflect its competing abilities. It was experimentally shown that, at least under laboratory conditions, if maintained together at a high density, larval survival of *An. maculipennis* is superior to that of both *An. messeae* s.l. and *An. beklemishevi* [36]. A similar situation can be expected to occur in natural habitats. Therefore, in cases of natural co-habitation, despite a high tolerance of *An. maculipennis* and *An. messeae* s.l. larvae to water composition (both are found in the brackish water of Lake Muldakkul), *An. maculipennis* can outcompete under optimal combination of other abiotic factors. However, the species that loses in the larval competition is still able to use atypical habitats with extreme conditions. Thus, in the waters of drainage canals from rice fields in Yablonovsky village (Ciscaucasia, a region where *An. maculipennis* is predominant), characterized by high saprobity and a high density of amphibians, only *An. messeae* s.l. larvae, although at an extremely low density, were found. In addition, abiotic factors, which can compensate for inferior survival of a species at the larval stage by increasing its survival at the imago stage, may favor species coexistence in certain territories. An increase in the proportion of *An. maculipennis* in the southwest and south, generally coinciding with the isotherms of the warm time of the year and shorter periods of cold weather, indicates the primary role of temperature (through both direct and indirect effects) in determining the outcome of interactions between the species. This is in agreement with an idea proposed by Ushakov [37], according to which, two closely related species are characterized by discrete differences in the thermal stability of cells, in line with the temperature of their habitat. In other words, speciation is usually associated with adaptation at the cell rather than organismal level. Indeed, malaria research institutions reported, using direct measurements, that *An. maculipennis* females start leaving their wintering eustatic shelters at an average daily temperature of 7.1°C [38], while for *An. messeae* s.l*.* this index was 4–5.0°C [39, 40]. Discrete differences between species in their reaction to temperature, in particular, a later outfly from wintering shelters of *An. maculipennis* females, delay the start of reproduction phase and apparently shift generations of species relative to each other. In the samples, this shift would manifest as an increase in the proportion of one of the species.

In line with temperature trends in northern Eurasia over the last 40 years, the distribution area of *An. maculipennis* s.s. was likely to have been expanding to the northeast previously as well, as was reported in 1989 [34]. However, this process was not accurately recorded at its early stage. Up to 1980s, when *An. maculipennis* s.s. was detected in Rostov, Suzdal, Murom, Yoshkar-Ola, Zelenodolsk and Samara using cytogenetic approach [34], the species was not observed in the region. Systematic studies carried out in Miass documented the emergence of *An. maculipennis* s.s. in the region. In 1999 and 2000, representative samples of *Anopheles* mosquito larvae collected in two ecologically distinct reservoirs in Miass were free from *An. maculipennis* (Table 1). In 2010, 2011 and 2012, several specimens from larval samples collected in these locations were identified as *An. maculipennis*. Despite the frequency of *An. maculipennis,* larvae are still low (up to 7% in some samples and absent in others), this species was found in a number of ecologically distinct habitats and in different years, therefore, its presence in Miass and consequently in the South Urals is now confirmed. The study of samples collected in Lake Muldakkul and in Koltashi village in 2012 revealed the presence of *An. maculipennis* in the first location and absence in the second, where *An. beklemishevi* was found.

Of note, in Miass, *An. maculipennis* was first identified in 2010 with a frequency similar to that reported for the northeast periphery of this species area in 1986–1987. Its low frequency in different biotopes suggests that it most likely emerged in Miass in 2008 or 2009. This allows estimating the rate of *An. maculipennis* expansion to the east.

Given that the time of sample collection in the eastern locality for the earlier study (Samara, City Park, 1986) and the most probable time when *An. maculipennis* appeared in Miass are separated by 22 years and about 750 km, and assuming that the border of the distribution area at that time was already shifted to the east of Samara, the average speed of the border movement eastwards is about 30 km per year. If true, this speed is significantly higher than estimated for other insect species, − 16.9 km per decade [24]. Because *An. maculipennis* is an endophilic species [41, 42], its expansion involves migration between populated places. Its endophilic nature and the possibility of passive mosquito migration in cars, trains and aircrafts is the most likely cause of rapid expansion of its distribution area. The territories between the Volga and the Southern Urals, although likely in patches, have already been invaded by *An. maculipennis*, and the vector of its expansion is from urban areas to the countryside. Taking into account the key role of temperature in the life cycle of mosquitoes and isothermal patterns in the European part of Russia and the Urals, the northern border of the *An. maculipennis* habitat is likely to move at the same speed, about 600–700 km further to the north of the border reported in 1986–1987 [34]. Moskaev (2012) [22] and Perevozkin *et al.* (2012) [28] found *An. maculipennis* s.s. in Karelia up to the town of Kem’ (64°57′N; 34°36′E). However, the distance between Rostov, where *An. maculipennis* at low frequency was discovered in 1986, and Kem’ is over 1000 km. In the literature, there is also a discrepancy in the description of the species composition in Kem’, where, according to Moskaev [22], all three species are present, while Perevozkin *et al.* [28] found only *An. maculipennis* and *An. beklemishevi*. Neither Stegnii *et al.* [14], nor Moskaev [22], nor the authors of the present study have detected the coexistence of *An. maculipennis* and *An. beklemishevi* in the absence of *An. messeae* s.l. in any of the geographical locations studied. Based on the data available so far, it can be assumed that the probable northern border of *An. maculipennis* habitat lies at a latitude of 64°N, i.e. to the north of the town of Segezha.

Species-specific responses of adult mosquitoes to temperature regimens [38–40], species competition at the larval stage of development [36], and patterns of their geographical distribution [15, 16, 41] suggest that the expansion of *An. maculipennis* to the north and east is a part of the biocenotic process caused by global warming. Changes in the borders of distribution areas and/or proportions of sibling species in the zones where the species are sympatric are also components of this process. The rate of climate warming has been estimated at 0.5°C per 100 years in the northern parts of European Russia and 1.4-1.6°C per 100 years in the south of the Urals, with the largest incremental changes in the past several decades [3]. One consequence of climate warming is an extension of reproductive period and shortening of hibernation period for mosquitoes. Thus, more thermophilic and less vulnerable *An. maculipennis* is given a better opportunity to invade the regions adjacent to the northern and eastern borders of its current distribution area. Expansion of *An. maculipennis* to the northeast is accompanied by a decrease in *An. messeae* s.l. and *An. beklemishevi* frequencies and probably even disappearance of the latter species. *An. beklemishevi* was found in the surroundings of Priozersk, Syktyvkar and Chelyabinsk in 1975 [14]. However, in the present study this species was absent in the samples collected in 1981 and 1999 in the region of Chelyabinsk (Kundravy, Miass) (Table 1). It should be noted that the samples from Kundravy village consisted of females in diapause, among which specimens of the exophilic *An. beklemishevi* could be absent due to environmental and behavioral characteristics of this species. Moskaev (2012) [22] did not find *An. beklemishevi* in Priozersk and confirmed its lower frequency in Syktyvkar in 2010 compared to the report of Stegnii *et al.* in 1978 [14]. Previously noted disappearance of *An. beklemishevi* in Zelenodolsk coincided with the emergence of *An. maculipennis* [34]. Steady climate change and foreseeable responses of the species studied allow predicting future events. Considering that most settlements to the east of the Urals are situated along the Trans-Siberian Railway and taking into account the endophily of *An. maculipennis*, this species will expand to the east within borders very similar to that of *An. messeae* A [27], i.e. as a gradually narrowing stripe of 300–400 km wide, with the borders lying in the south of the forest and the north of the forest-steppe zones. Obviously, the speed of a species expansion will depend on the temperature dynamics in the region. It is important to evaluate possible consequences of the growing degree of overlap between the distribution areas of *An. maculipennis* and both cryptic species of *An. messeae* s.l., as well as increasing contacts between *An. maculipennis* and *An. beklemishevi*. At the larval stage, under conditions of high-density, *An. maculipennis* demonstrates superior survival compared to *An. messeae* s.l., i.e. both A and B species [36]. *An. messeae* A and *An. maculipennis* are considered as endophilic species, while *An. messeae* B is exophilic [20]. Therefore, *An. maculipennis* and *An. messeae* A have very similar environmental needs, and their ecological niches largely overlap, leading to their competition and as a result, reduction in the frequency of the latter species. Indeed, cytogenetic analysis showed that *An. maculipennis* emergence in Miass was accompanied by a change in the proportions of the *An. messeae* cryptic species. In line with this finding, we detected a highly significant (p < 0.001) decrease in the frequencies of homozygotes, heterozygotes and hemizygotes for inversion 1L1, as well as other cytogenetic markers of *An. messeae* A, over the period from 1999 to 2011, accompanied by a corresponding increase in the frequencies of markers typical for the *An. messeae* B (Table 2). In addition, a reliable cytogenetic marker of *An. messeae* B, the inversion 1L2 on the sex chromosome, reached a detectable frequency in the region. The 2011 sample from location ‘Miass-1’ contained 2 heterozygous females and 1 hemizygous male for this inversion out of 76 *An. messeae* s.l. specimens. Proportions of the A and B species and frequencies of marker 1L chromosome variants in *An. messeae* A were determined in the samples collected in 2012 using ITS2 PCR-RFLP (Table 3). Species identification was performed for 102 *An. messeae* s.l. specimens (32 females and 24 males of *An. messeae* A and 28 females and 18 males of *An. messeae* B), for these the presence of the inversions on X-chromosome was also determined. Only one male, identified by ITS2 PCR-RFLP as belonging to *An. messeae* B, had variant 1L1/¬. Thus, the inversion 1L1 is highly specific for *An. messeae* A (provided that the inversions 1L1 are identical in both cryptic species, and the latter not fully proven yet [27]). Thus, a significant increase in the proportion of *An. messeae* B (p < 0.01) from earlier to later samples, as defined based on marker inversion variants, reflects the actual sequence of events. This allows us to estimate the proportions of *An. messeae* cryptic species A and B by analyzing the frequencies of the above cytogenetic markers in other regions and locations. Analysis of species proportions in different geographic locations (Table 2) showed an increase in the frequency of cytogenetic markers typical for *An. messeae* B in those instances when *An. maculipennis* simultaneously occurs in this area or when its frequency is increased.

**Table 3:** **The composition of chromosome arm 1L variants in** ***An. messeae*** **A and B (after species identification of larvae by ITS2 PCR-RFLP; Miass, 2012)**

Species	***An. messeae***A		***An. messeae***B		***An. messeae***s.l.	
Variants	N	f (%)	N	f (%)	N	f (%)
1L/1L	16	50.0 ± 8.8	28	100	44	73.3 ± 5.7
1L/1L1	11	34.4 ± 8.4	0	0	11	18.3 ± 5.0
1L1/1L1	5	15.6 ± 6.4	0	0	5	8.3 ± 3.6
Total females	32	100	28	100	60	99.9
1L/¬	16	66.7 ± 9.6	17	94.4 ± 5.4	33	78.6 ± 6.3
1L1/¬	8	33.3 ± 9.6	1	5.6 ± 5.4	9	21.4 ± 6.3
Total males	24	100	18	100	42	100
Total individuals	56	54,9 ± 4,9	46	45,1 ± 4,9	102	100

Note: N – sample size; standard deviation is given as a measure of statistical error.

For example, in Solnechnogorsk, Suzdal, and Protvino where the proportion of *An. maculipennis* is high or it is dominant over other species, the frequency of the cytogenetic markers typical for *An. messeae* B is relatively high. However, in areas where *An. maculipennis* is absent or rare, the proportion of *An. messeae* A is increased, which is signaled by high frequencies of chromosome variants typical for this species (Rybinsk, Yaroslavl and Rostov). In some regions this correlation is not observed (Yoshkar-Ola), which is likely due to the low numbers of *An. messeae* A individuals in this region. In Ciscaucasia and in the suburbs of Kharkov, in accordance with its cytogenetic structure, only *An. messeae* A was found in conjunction with *An. maculipennis*. In Khadyzhensk and in the suburbs of Kharkov, the latter species was predominant (Table 2). Despite significant (p < 0.05) differences in the proportions of species between the samples from Khadyzhensk and Kharkov, differences in the frequencies of 1L/1L1, 1L1/1L1 and several other variants were not detected. Thus, in the competition with other species, *An. messeae* A behaves as a whole, without showing any significant advantages for carriers of specific inversions and their combinations. This means that cytogenetic changes of *An. messeae* s.l. larvae in Miass, which occurred after the appearance of *An. maculipennis*, were due to a change in the frequencies of these species rather than a change of inversion frequencies in the cryptic species. A finding which was unexpected and will require further studies was a decrease in the proportion of *An. messeae* A in Miass given a low presence (up to 7%) of *An. maculipennis*. It is also possible that the observed trends in species frequencies result from not only competition between *An. maculipennis* s.l. species*,* but also from alterations in other ecosystem components. Our results describing the impact of *An. maculipennis* on the ratio of *An. messeae* species A and B are consistent with the situation described in Germany, where *An. messeae* s.l. and *An. maculipennis* are sympatric. According to the results of sequencing of marker portions of genomic DNA from mosquitoes collected in localities with low (a few percent) and high (over 50%) occurrence of *An. maculipennis*, *An. messeae* species referred here as the species B, outnumbered the other one [43].

Possible consequences of *An. maculipennis* expansion to the Urals, and likely, soon after that, to Western Siberia can be foreseen. In parallel with a decrease in the number of *An. messeae* s.l., the proportion of *An. messeae* A cryptic species will also decline, together with a respective increase for *An. messeae* B. The latter will manifest itself through increasing karyotype variability, i.e. a process opposite of that previously described [20]. Thus, *An. maculipennis* will follow the path of colonization of Siberia pursued earlier by *An. messeae* A, which is characterized by similar environmental needs but is somewhat more resistant to low temperatures. Expansion of *An. maculipennis* to the north and east will continue until an equilibrium is achieved among the four species, two of which (*An. maculipennis* and *An. messeae* A) are endophilic and thermophilic and the other two (*An. messeae* B and *An. beklemishevi*) are exophilic and cold-resistant. This balance will be maintained mainly due to different durations of the reproductive period typical for each species, during which a growth in the frequencies of the first two species is expected, and this growth will be more pronounced for *An. maculipennis*. The second factor to play a role will be the availability of wintertime eustatic shelters and temperature in those during the cold time of the year, when the numbers of the first two species will decline more rapidly. These conditions will be less severe for a more cold-resistant *An. messeae* A compared to *An. maculipennis*. Expansion of *An. maculipennis* is likely to be accompanied by ousting of *An. beklemishevi* from more or less populated areas, and correspondingly, the southern and western borders of the distribution area of the latter species will move to the north and east. However, because *An. beklemishevi* is exophilic, it will still be able to survive, although in small quantities, in the wild. Given that *An. maculipennis* is able to transfer *Plasmodium vivax,* and that global warming leads to increased species diversity and abundance of *Anopheles* on large territories, one can confirm a worsening epidemiological situation in Eurasia.

**Figure 1:**
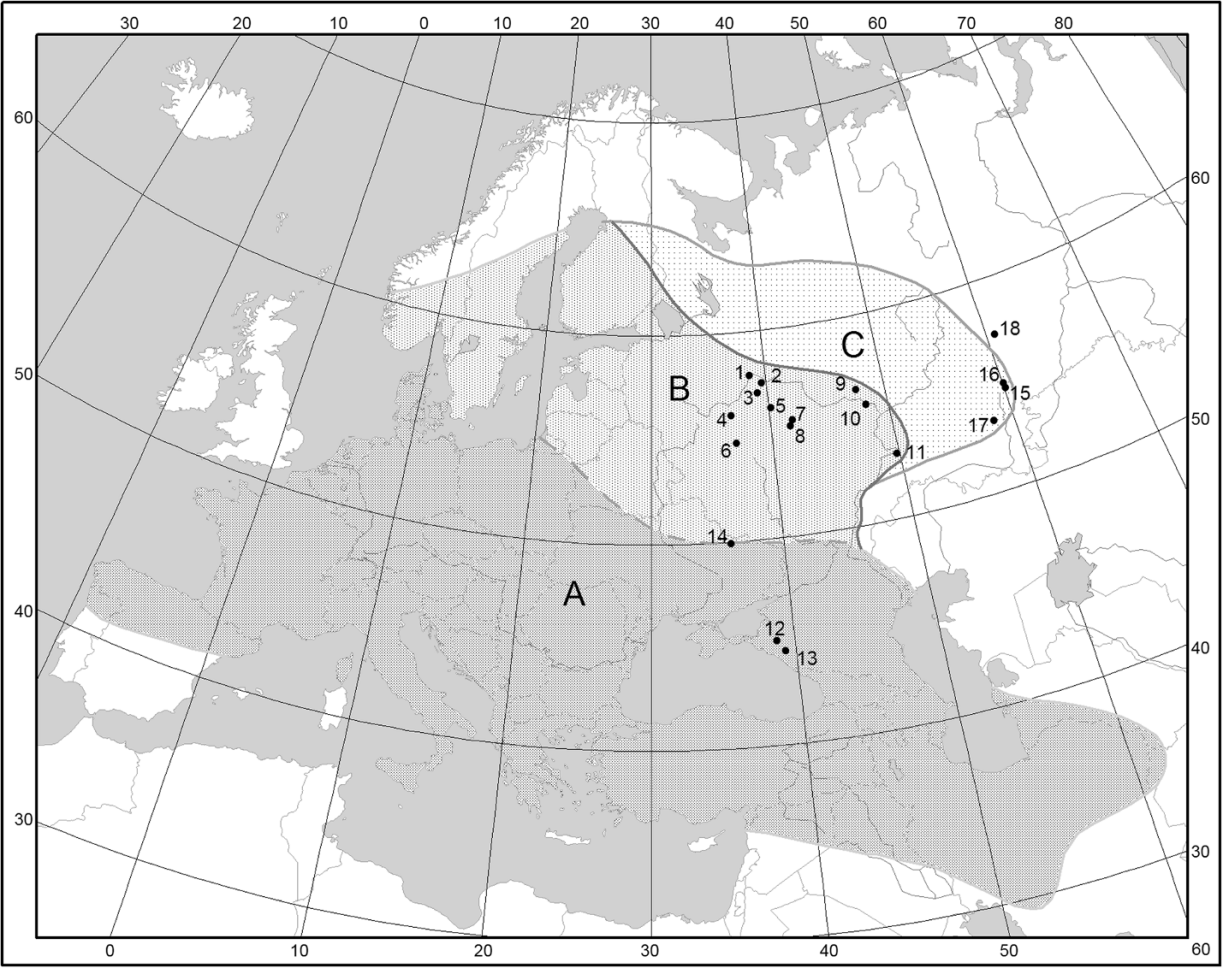
**Dynamics of** ***An. maculipennis*** **distribution area: A –** ***An. maculipennis*** **distribution area described by White (1978) **[15]**, B – by Novikov, Alekseev (1989) **[34]**, with the additions by Ramsdale and Snow (2000) **[45]** for Scandinavia and Ciscaucasia; C – the expansion of the area from 1986 to 2010.** Locations have the same numbers as in Table 1.

Results of the present study and previously published data on *An. maculipennis* geographic and temporal distribution made it possible to reconstruct the dynamics of the area occupied by the species (Figure 1). White (1978) [15] and Novikov & Alekseev (1989) [34] reported conflicting data on the northeastern border of its area (segment B in Figure 1). This can be explained by the expansion of *An. maculipennis* to the northeast and by the limited information available at the time White’s review was prepared. Our results favor the former explanation for this discrepancy. Expansion of the distribution area during the period from 1986 to 2010 (segment C in Figure 1) can be ascertained based on our results and the results of other researchers [22, 28]. Due to lack of detailed data on *Anopheles* distribution in Scandinavia [44], the borders of its distribution area in this region were determined based on data provided by Ramsdale & Snow [45]. The southeastern borders reported by White (1978) [15] have been confirmed by recent studies. In Iran, *An. maculipennis* was found from the southeastern shore of the Caspian Sea to settlements located to the northeast of the Persian Gulf [46]. *An. maculipennis* was also found in Turkey, Iraq and Syria, but not in Tajikistan and Afghanistan [47, 48].

## Conclusions

Significant increase in the global temperature, i.e. global warming, recorded in the past 40 years, has resulted in the changes of Maculipennis Complex species distribution in Eurasia. The distribution area of *An. maculipennis* s.s. is expanding to the northeast at an average speed of approximately 30 km per year; in 2008–2009 the species appeared in the Southern Urals. Colonization by *An. maculipennis* s.s. leads to a decrease in the frequency of *An. messeae* s.l. and its cryptic species *An. messeae* A, and a concomitant increase in the frequency of *An. messeae* B. Likely, this is also accompanied by either active gradual ousting or passive disappearance of *An. beklemishevi*. Ecologically, the above species, particularly *An. maculipennis* and *An. beklemishevi*, primarily differ in their responses to temperature. The latter is manifested in diversification of species over time, different rates of development during reproductive periods and differential resistance to low temperatures during overwintering. The ecological niches of *An. maculipennis* and *An. messeae* A most considerably overlap, while for those of *An. maculipennis* and *An. messeae*-B only a minor degree of overlap is observed. Ecologically, *An. maculipennis* and *An. beklemishevi* are very similar. However, they are sharply demarcated with respect to certain abiotic factors (mineral composition and temperature of water) whose dynamics would significantly modify the effect of all other factors. In the northeastern periphery of the *An. maculipennis* distribution area, sympatry with *An. messeae* s.l. is compulsory, with *An. beklemishevi* – possible; coexistence of *An. maculipennis* and *An. beklemishevi* in larval habitats in the absence of *An. messeae* s.l. is an exception. On this territory, one can expect cyclic fluctuations both in the number and the frequency of *An. maculipennis*, with ups during the warm (reproductive) season and downs during wintertime.
